# Evaluating the impact of a pilot social prescribing service for the armed forces community

**DOI:** 10.1017/S1463423626100875

**Published:** 2026-02-24

**Authors:** Mariyana Schoultz, Lori Boul, Emma Senior, Amy Swift, Matthew D. Keirnan

**Affiliations:** 1 Northumbria University - Coach Lane Campus: Northumbria University Faculty of He, UK; 2 Northumbria University, UK

**Keywords:** Armed forces, evaluation, link-worker, mixed methods, social prescribing, veterans, wellbeing

## Abstract

**Aim:**

This study evaluated the impact of a novel social prescribing service designed specifically for the Armed Forces Community (AFC) and its influence on service users’ wellbeing.

**Background:**

Social prescribing connects individuals with non-clinical, community-based support to address loneliness, long-term conditions, and mental health. Despite advances in social prescriber training, a gap remains in resources for working with the AFC, who present distinct wellbeing needs. A two-year project, funded by the NHS Armed Forces Health and the Armed Forces Covenant Fund Trust, sought to enhance provision by equipping Social Prescriber Link Workers with specialist skills.

**Methods:**

A sequential mixed-method design was adopted. Quantitatively, changes in wellbeing for 259 AFC service users were measured using the Short Warwick and Edinburgh Wellbeing Scale before and after consultations with Armed Forces Social Prescriber Link Workers (AFCSPLWs). Qualitatively, semi-structured interviews were undertaken with AFCSPLWs, general social prescribers, General Practitioners, and AFCSPLW line managers to explore experiences of service delivery.

**Findings:**

Wellbeing scores significantly increased from initial (mean = 15.3) to final consultations (mean = 18.79), indicating positive effects; however, scores remained lower than national averages, reflecting the complex needs within the AFC population. Thematic analysis identified four themes: Armed Forces Experience and Perspective, Challenges and Barriers, Service Delivery and Effectiveness, and Skill Development and Attributes. Subthemes highlighted AFC-specific challenges, the practical complexity of the AFCSPLW role, and the importance of cultural competence. Advocacy, navigation, and relationship-building emerged as central mechanisms, with AFCSPLWs acting as vital connectors across primary care, third-sector services, and professional networks.

## Background

### Evolution of social prescribing in healthcare

Social prescribing has long been a part of healthcare, with referral programs akin to social prescribing existing in the UK for over 30 years. However, the formal integration into the NHS and the structured development of social prescribing initiatives is relatively recent, significantly gaining momentum post-2006 with the publication of the white paper ‘Our Health Our Care’ (Kings Fund, 2017). Social prescribing has been shown to reduce demand on primary and secondary care services, improve mental health and well-being, and enhance social connectedness. Studies have reported positive outcomes such as reduced GP consultations, decreased hospital admissions, and improvements in patients’ quality of life and mental health (Polley *et al*., 2017; Husk *et al*., 2019).

### Models of social prescribing

Kimberlee (2015) categorizes social prescribing into several models, each with distinct features and implementation strategies: Broker Model, Direct Referral Model, Community Hub Model, and Signposting Model. The social prescribing model being evaluated in our study aligns closely with the Broker Model. This model involves dedicated Armed Forces Social Prescriber Link Workers (AFCSPLWs) who work closely with AFC service users to identify their unique needs and connect them to tailored community resources and services. The AFCSPLWs act as intermediaries, ensuring that the services provided are culturally competent and specifically designed to address the psycho-socio-cultural differences between military and civilian life.

### Standard vs. AFC-specific social prescribing practices

Standard social prescribing practices are primarily designed to address the needs of the civilian population. However, members of the Armed Forces Community (AFC) are a minority group with specific needs that differ significantly from those of civilians. Understanding the psycho-socio-cultural differences between military and civilian life and the challenges of transitioning to civilian life is crucial for effective support delivery (Demers, 2011; Ray, 2011; Stachyra, 2011; Banks, 2018). While generic training courses address issues related to diversity and minority groups, they often lack specific training and resources for working with the AFC.

### Developing skills and knowledge for the armed forces community (AFC)

Some organizations, such as the NHS e-Learning Healthcare for the Armed Forces, the Armed Forces Covenant Fund Trust, the Soldiers’, Sailors’, Airmen’s Families Association (SSAFA), and Combat Stress, provide training courses covering partnership working with veterans’ health, veterans’ rights, signposting, and understanding the broader determinants of health and well-being. These courses offer valuable information on practical support for needs affecting the health of AFC service users, such as housing, employment, and legal issues (Bertotti *et al*., 2019), as well as local voluntary and community enterprises offering appropriate support (Fixsen *et al*., 2020). However, social prescribing link workers require not only a broad range of knowledge and understanding of biopsychosocial and emotional determinants of health but also cultural competence. The Workforce Development Framework: Social Prescriber Link Worker (NHS, 2023) includes cultural competence, highlighting the importance of working effectively with individuals from different cultures. The Personalised Care Institute Curriculum (2020) refers to groups protected by the Equality Act 2010, such as BAME and LGBT communities, as well as vulnerable migrants and individuals residing in areas of socio-economic deprivation. However, it has been recognised that service providers, who are predominantly white and middle class, may lack the proximity and understanding of the complexities faced by Black, racial minority, and AFC (Durrant and Rolston, 2022). Similar to other forms of cultural competence, understanding military culture is essential for healthcare professionals to better support individuals with military experiences (Westphal and Convoy, 2015).

### Pilot study and demonstrator project

The transition period for individuals leaving the military is recognized as a critical phase, making social prescribing during and after this period particularly valuable. The AFC comprises a diverse group of individuals in terms of age, gender, sexual orientation, socio-economic background, nationality, and ethnicity. While members of the AFC face similar practical difficulties as civilians, such as housing, finances, employment, and mental health challenges (Veterans Gateway, 2024), they often experience these difficulties in unique ways due to factors related to military life (Kiernan *et al*., 2018; Wilson *et al*., 2018; Gettings *et al.*, 2022; Osbourne *et al.*, 2022). Thus, in addition to addressing practical issues, understanding the psycho-socio-cultural differences between military and civilian life is crucial for effective support delivery (Demers, 2011; Ray, 2011; Stachyra, 2011; Banks, 2018).

### Research gap

Despite the increasing recognition of social prescribing’s efficacy, there remains a dearth of research specifically addressing the skills, training, and factors influencing its implementation for the AFC. This was confirmed by our literature search conducted to identify relevant studies and literature on social prescribing and the Armed Forces Community. The search encompassed peer-reviewed articles, policy documents, guidance, and grey literature. The search was limited to articles published in English between 2018 and 2023. Various search terms were used, including generic terminology related to social prescribing and specific terms related to the military and veterans (e.g., ‘social prescribing’, ‘community navigator’, ‘health trainer’, ‘care navigator’, ‘link worker’) and specific terms (e.g., ‘military’, ‘veterans’, ‘armed forces’, ex-military, ex-forces). Synonyms and reference lists from previous reviews and meta-analyses were also consulted. The searches revealed a wealth of literature available on various issues related to Social Prescribing, e.g., evaluation, cost, monitoring, health and mental health conditions, loneliness, suicide and community activities (i.e., sports, art, gardening etc.). Despite an abundance of literature on social prescribing (*n* = 3014) covering various topics, only one of the articles reviewed specifically addressed social prescribing and the AFC (Mottershead, 2022). Of the articles and reports specifically focused on the Armed Forces (*n* = 65) two contained brief references to Social Prescribing (Bacon *et al*., 2021; Steen *et al*., 2021). This gap highlights the lack of published research on skills, training, and factors influencing the implementation and delivery of social prescribing for the AFC, despite their unique health and well-being needs.

A pilot study, conducted in Durham/Dales GP practices, highlighted some significant challenges and knowledge and skill gaps in relation to working with AFC service users (Kiernan *et al*, 2019). Recognizing the opportunity to improve the roll-out of social prescribing, a 2-year demonstrator project was funded by the NHS Armed Forces Health Team and Armed Forces Covenant Fund Trust. The project, managed by the South West Integrated Personalised Care Team and Northumbria University’s Northern Hub for Veteran and Military Families’ Research, aimed to equip Social Prescribers with the knowledge and skills necessary for working with the AFC. The project included the development of bespoke software, recruitment of dedicated Armed Forces Social Prescriber Link Workers (AFCSPLWs), the launch of an online introductory educational module, and the creation of an educational package for AFCSPLWs to deliver training to Social Prescribers. The demonstrator project sought to establish a replicable model that can be scaled across the UK. Having been in operation for two years, it is now poised to evaluate the impact of the work carried out by AFCSPLWs. This evaluation provides valuable insights into the effectiveness and outcomes of the project, ultimately contributing to the advancement of social prescribing services for the AFC.

## Methods and materials

### Design

This study utilized a sequential mixed-method design, combining quantitative and qualitative data analysis. The quantitative component involved administering a validated questionnaire to assess changes in well-being scores before and after appointments with AFCSPLWs. The qualitative component entailed conducting semi-structured interviews to explore stakeholders’ experiences and perspectives on the implementation and use of the AFCSPLW model.

### Participant and recruitment

A convenience sample was recruited from service providers and users engaged in AFC networks. These included a diverse range of stakeholders involved in social prescribing: AFC social prescribers, general social prescribers, GPs and AFCSPLW Line Managers. Recruitment involved reaching out to potential participants in the selected areas in UK and providing them with information about the study. Voluntary participation was invited, and interested individuals were provided with consent forms and information sheets detailing the study’s purpose and procedures. Prior to their involvement, written consent was obtained from each participant.

### Referrals

Individuals were referred to the social prescribing service based on a variety of factors, which align with the broader objectives of promoting health and well-being within the community. While the specific focus of our study is on the AFC, the reasons for referral into the service may mirror those observed in the general population. Common reasons for referral include, but are not limited to: employment (23.33%), Mental Wellbeing (16.97%), Housing 11.89%, Finances 11.70%’ COVID-19 Local Support 10.32%, Physical Health 9.02%, Family and Communities 6.36% (Veterans Gateway, 2024)

### Materials

For the quantitative component, the Short Warwick and Edinburgh Wellbeing Scale (SWEMWBS-7 item) was utilized (NG Fat, 2017). This validated questionnaire was administered by AFCSPLWs to service users at the initial and final appointments to assess changes in well-being. The well-being scores obtained from the SWEMWBS were then recorded and submitted to the researcher for further analysis. In conjunction with the quantitative assessment, sessions with AFCSPLWs were tailored to individual needs and preferences. AFCSPLWs aimed to establish rapport, identify the needs, and collaboratively develop personalised care plans. Discussions encompassed various aspects of service user lives, including health concerns, social connections, and community resources. Although not adhering to specific techniques, AFCSPLWs employed principles of empathy and active listening to foster a supportive environment conducive to positive change and improved well-being.

In the qualitative component, semi-structured interviews were conducted to gather insights into participants’ experiences with the AFCSPLW model. Interview guides were developed to ensure consistency across interviews while allowing flexibility for participants to elaborate on specific topics. The interviews were audio-recorded with participants’ consent and transcribed for analysis.

## Data analysis

### Quantitative

Quantitative data collected from the SWEMWBS questionnaire underwent statistical analysis using the SPSS v.27 software. Descriptive statistics and inferential analyses. Specifically, a paired samples t-test was conducted to compare participants’ well-being scores at the initial and final consultations

### Qualitative

The qualitative data from the interviews was analysed using a thematic analysis approach. We followed the six step approach (Braun *et al*., 2021). The first step involved familiarising the researchers (MS, AS, and ES) with the qualitative data collected from the semi-structured interviews. This process included repeated readings of the interview transcripts to gain a comprehensive understanding of the content. After gaining familiarity with the data, the researchers generated initial codes. Line-by-line coding of the transcripts was conducted to identify meaningful units within the data. Each meaningful unit was assigned a descriptive label or code. Next, the researchers searched for themes within the initial codes, reflecting meaningful aspects of participants’ experiences and perspectives. Similar codes were grouped together to form potential themes. In step four, the researchers reviewed the potential themes and checked their coherence and relevance to the research questions and data. They examined whether the themes accurately represented the participants’ experiences and perspectives. The researchers then reviewed and refined the themes naming each theme to concisely capture its essence. The final step involved analysing relationships between themes and subthemes. The researchers explored how different themes were interconnected and how they contributed to the overall understanding of the research topic. Any emerging patterns or relationships between themes were carefully examined and discussed.

## Ethical considerations

Ethical approval was obtained from Northumbria University with reference number NU0427 prior to data collection. The study therefore adhered to ethical guidelines, ensuring participant confidentiality, informed consent, and protection of personal data.

## Results

### Quantitative results

A total of 259 service users completed the SWEMWBS measure at two time points: when they were first referred to the service (initial consultation) and again when the support ended (final consultation) between 1 Oct 2021–31 Jan 2023. It is important to note that while 259 service users provided matched baseline and follow-up data, information on the total number of individuals who completed only the baseline measure is not available. Therefore, attrition rates cannot be calculated, and the representativeness of the matched sample relative to all service users remains unclear. Additionally, without comparative demographic data for the entire population served by the service, it’s challenging to determine if the sample adequately reflects the gender and age distribution of all service users. Furthermore, while the data suggests no attrition between baseline and follow-up assessments, the absence of explicit information on attrition rates throughout the study limits our understanding of participant retention. Brief demographic data is presented in Table 1.

**Table 1.** Demographic data for AFCSPLW service users

Characteristics	***N***	%
No of participants	259	100
Age range	24–95	n/a
Sex		
F	25	9
M	234	91
**Type of service**		
Army	164	63
Navy	44	17
Royal air force	35	14
Royal marine	5	2
Royal marine-reserves	1	0
Other	10	4
**Status**		
EX military	247	95
Family	2	.8
Spouse	6	2.3

### Referrals

The study has received referrals predominantly from a specific geographical area in Central Cornwall. Initial referrals were primarily facilitated by Social Prescribing Link Workers, comprising 61.5% of the total referrals, as per Table 2. However, as the project progressed, promotion efforts by AFCSPLWs expanded referrals from other sources. The main reason for referrals was social isolation, accounting for 28% of primary referrals and frequently noted as a secondary concern. Other common reasons included mental health issues, physical health concerns, financial difficulties, and housing challenges, as outlined in Table 3. Support provided predominantly involved signposting, which constituted 58% of actions taken, followed by one-to-one support for 28% of clients. The duration of one-to-one support varied depending on individual needs, typically lasting 2 to 4 weeks (Table 4). While professional advice appeared lower than expected, it’s worth noting that these figures do not encompass training sessions, initial meetings with healthcare professionals, or ad hoc telephone consultations, which often provide valuable guidance within a short timeframe.

**Table 2.** Referrals

Source of referral	%
Social prescribing link worker	61.5
Other	19
Social care	7
Volunteer charity sector	6
Self	6
GP	.5

**Table 3.** Reasons for referrals

Reasons for Referral	Primary %	Secondary %
Social isolation	28	13
Mental health	14	1
Physical health	13	3
Finances i.e., benefits	18	18
Housing	9	0
Employment	5	1
Other	13	11

**Table 4.** Support/action

Support provided	%
Professional advice	14
Sign posting	58
One to one support	28

### Service user wellbeing

We used a scale called the Short Warwick and Edinburgh Wellbeing Scale to measure the well-being of the participants. The scale gives a score that represents a person’s well-being, with higher scores indicating better well-being. The participants completed the scale at both the initial consultation with the AFCSPLWs and the final consultation.

The results indicated a statistically significant improvement in well-being from the initial consultation (*M* = 15.03, *SD* = 3.66) to the final consultation (*M* = 18.79, *SD* = 4.17), with a mean difference of 3.76 (*t*(258) = 30.7, *p* < .001). Table 5 displays the descriptive statistics of the first and second SWEMWBS scores, demonstrating a 25.01% increase in well-being. This analysis provided evidence of the positive impact of the support provided by AFCSPLWs on the well-being of participants. However, the mean scores at both consultations were considerably lower than the scores found in a national health survey conducted in 2011 (NHS Digital, 2012). This indicates that the individuals who sought support from AFCSPLWs had more complex health and social care needs compared to the general population.

**Table 5.** Difference between the average first and second scores (*N* = 167)

	1st SWEMWBS	2nd SEMWBS	Difference	Percental difference
Mean	15.03	18.79	3.76	25.01%

### Qualitative results

Semi-structured interviews were conducted with 11 stakeholders (six AFCSPLWs, four Managers and one GP) in order to gain multiple perspectives on their experiences of the implementation and on-going use of the model. Four main themes were identified with ten corresponding subthemes capturing different nuances. The four main themes were: Armed Forces Experience and Perspective Challenges and Barriers, Service Delivery and Effectiveness and Skill Development and Attributes. Please see the corresponding subthemes in Table 6.

**Table 6.** Themes and subthemes from stakeholder interviews

Themes	Subthemes
Armed forces experience and perspective	Armed forces life
	Armed forces perspective
Challenges and barriers	Personal challenges
	Trust and attitude difficulties?
Service delivery and effectiveness	Accessing information and services
	Service quality and factors
	Involvement and collaboration
Skill development and attributes	Advocacy and support
	Building relationships and rapport
	Project management

#### Theme 1: armed forces experience and perspective

The first theme is essential in understanding how the unique journey of individuals who have served in the Armed Forces influences their engagement with the Social Prescribing Link Worker (SPLW) service. By exploring their experiences and perspectives, we gain valuable insights into how social prescribing can be optimized to address the specific needs of this distinct community. The experiences shared by participants underscore the enduring impact of military service on individuals’ identities and social connections. The sentiment expressed regarding the camaraderie among service members highlights the significance of peer support networks within the Armed Forces community. These insights emphasize the need for social prescribing services to recognize and leverage the sense of camaraderie and shared experiences among veterans to foster engagement and support.
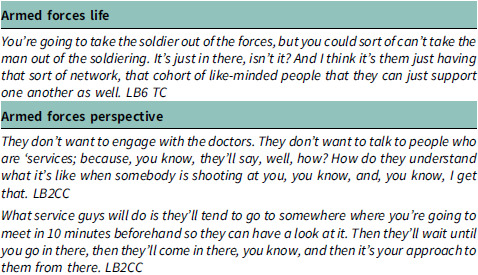



#### Themes 2: challenges and barriers

The second theme highlights the various hurdles that individuals may encounter when accessing and engaging with the Social Prescribing Link Worker (SPLW) service. Within this theme, the personal challenges faced by individuals, such as the emotional burden of sharing deeply personal experiences, highlight the complexities inherent in addressing the multifaceted needs of veterans. Additionally, the difficulties related to trust and attitude underscore the importance of building rapport and understanding within the social prescribing relationship. These findings underscore the importance of tailoring support approaches to address individual needs and preferences sensitively.
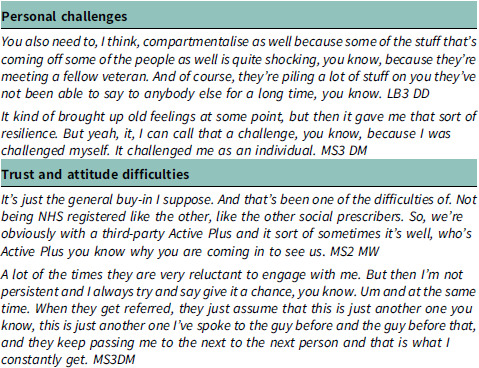



#### Theme 3: service delivery and effectiveness

This theme focuses onto critical factors influencing the delivery and effectiveness of social prescribing services. The positive reception from healthcare practitioners underscores the potential of social prescribing to complement existing healthcare services. However, challenges related to organizational recognition and funding highlight the need for greater integration and support within existing healthcare structures. Moreover, the emphasis on personalized care and the role of individuals with military backgrounds highlights the importance of cultural competence and shared experiences in facilitating engagement and trust.
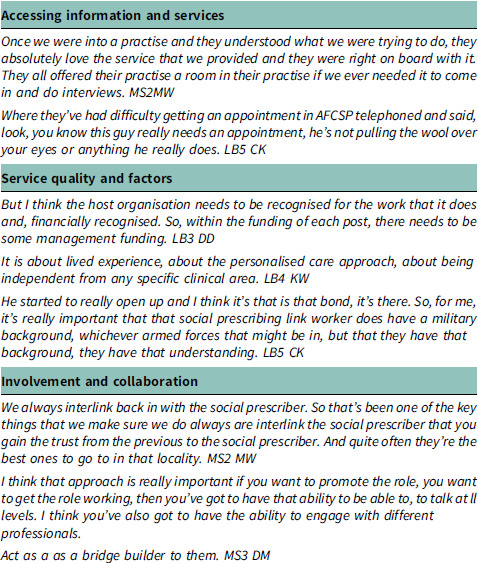



#### Theme 4: Skill development and attributes



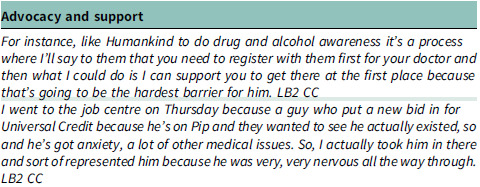





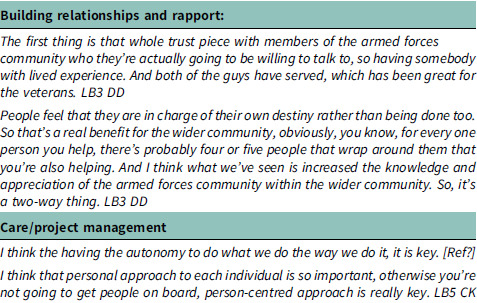



## Discussion

This study evaluated a novel pilot service, providing crucial insights into the impact of the Armed Forces Social Prescribing Link Worker (AFCSPLW) service on the well-being of its users. Its aims were to improve the health outcomes for the armed forces community which have a range of unmet needs (Bacon *et al*., 2021). The veteran population is a diverse group of 2.4 million people (Ministry of Defence [MoD] 2019), with over half of its population being aged 75 or older, and specific subgroups facing higher risks of mental illness, particularly those with combat experience or injuries (Forbes *et al*., 2011; Bergman *et al*., 2016; Osório *et al*., 2017).

Transitioning to civilian life can pose significant challenges for ex-military personnel, including employment difficulties, substance misuse issues, involvement with the criminal justice system, and various physical and psychosocial concerns related to their service (Hughes, 2017). To meet the needs of the AFC adequately, health providers and services must possess a deep understanding of this population’s unique requirements and be well-informed about their military culture.

While previous initiatives like ‘Fighting Fit’ and Combat Stress networks have aimed to support veterans, there has been a clear need for bespoke, Armed Forces-accredited services that deliver high-quality care (Murrison, 2010). This study offers promising evidence of the effectiveness of such services, and shedding light on key aspects of AFC-specific social prescribing services. Unique in its approach, the study captures the perspectives of stakeholders directly involved in delivering and receiving support from the service.

The quantitative findings underscore the potential of the AFC social prescribing service to address the multifaceted needs of veterans. Despite this positive change in wellbeing scores, it’s noteworthy that the AFC population still falls within the classification of low well-being, prompting further inquiry into the reasons behind this observation. Several factors may contribute to this phenomenon, including the complexity of the issues faced by AFC members, the potential need for more extensive support or longer-term interventions, and the multifaceted nature of well-being itself. The findings suggest that while the AFCSPLW service shows promise, there may be additional considerations to enhance its effectiveness fully. Nonetheless, the quantitative results align with the qualitative findings, which emphasize the significance of knowledge and cultural competence when linking service users with veteran-specific occupational engagements and enrichment activities, particularly for veterans who were previously challenging to engage or those experiencing higher levels of loneliness (Wendelton, 2019). Moreover, as over 85% of veterans are male, and research suggests that men may gain greater benefits from social prescribing (Woodall *et al*., 2018), the significance of these services for this demographic is evident.

The qualitative results, derived from semi-structured interviews with professionals involved in the AFC service, provided valuable context to the quantitative findings, shedding light on the nuanced experiences and perspectives of both service providers and their perceptions of users within the AFC community as well as providing multiple perspectives of the AFCSPLW model. One critical theme, ‘Armed Forces Experience and Perspective,’ highlighted how veterans’ unique military experiences influence their engagement with the service. Understanding this background enables AFCSPLWs to deliver culturally responsive support, fostering better engagement and outcomes.

The interviews also identified a diverse number of challenges and barriers from both service provision and service user perspectives. By acknowledging the challenges and barriers encountered such as personal challenges, mental health issues, physical disabilities, financial limitations, and trust and attitude difficulties, we gain a deeper understanding of the obstacles hindering optimal engagement with social prescribing services. While these barriers may have similarities in the general populations (Brandling, 2012), by acknowledging and addressing these challenges, with compassion, service providers can ensure personalized care that respects the unique circumstances of each individual. Addressing personal struggles and establishing trust with service user are key factors in enhancing the effectiveness of social prescribing interventions (Farenden *et al*., 2015). This approach ultimately results in improved well-being and health outcomes for those utilizing the service. Additionally, it helps us better understand the obstacles individuals may encounter when trying to access and engage with the AFCSPLW service. Some of the other challenges were related to the risk of continuity of the service due to third sector services funding is often cut and therefore hinders the delivery of continuous care. This aligns with the general social prescribing literature (Farenden *et al*., 2015; Pescheny, 2018).

The stakeholders identified effective service delivery as another theme of importance, relating to easy access to information and services, maintaining service quality, and fostering collaboration among stakeholders. This last aspect is notable, as effective relationships between the voluntary and community sectors and primary care services have been linked to the success of social prescribing interventions (Bickerdike *et al*., 2017). Previous studies as well as the interviews revealed that nurturing these relationships takes time (Bickerdike *et al*., 2017), but the quality of those relationships can be critical in the effectiveness of social prescribing interventions (South *et al*., 2008; Dayson, 2017).

Lastly, the final theme was related to skill development and attributes, emphasizing the multifaceted role of AFCSPLWs. These professionals not only provide services but also act as advocates, educators, mentors, care navigators and outreach workers. For veterans, who often face bureaucratic hurdles and fragmented services, having an advocate in their corner is invaluable. The AFCSPLW’s role in advocating for veterans, bridge gaps and streamline access to necessary resources. This subtheme underscores how AFCSPLWs are not just service providers but also powerful allies, making social prescribing interventions more effective and tailored to veterans’ needs. The ability to build meaningful relationships and establish rapport is another vital component of the AFCSPLW’s skill set. Veterans, due to their unique experiences and potential challenges such as post-traumatic stress or adjustment difficulties, may be initially hesitant to engage with healthcare and support services. In the context of social prescribing, building trust and rapport with veterans is a foundational step. Through these connections, veterans are more likely to engage with social prescribing interventions, knowing they have a supportive and empathetic ally guiding them toward improved well-being. The AFCSPLW’s role is multifaceted, encompassing coordination, planning, and execution of social prescribing interventions. These interventions can involve complex care plans, referrals to various community resources, and tracking progress over time, educating primary care, fostering relationships.

**Figure 1.**
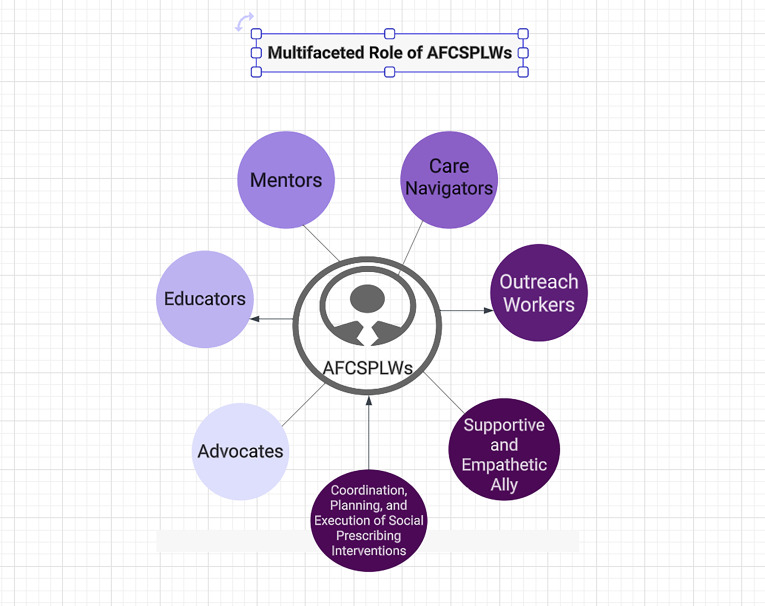
The multifaceted role of AFCSPLWs.

Overall, the findings of this study provide comprehensive insights into the multifaceted role of AFCSPLWs as advocates, educators, mentors, care navigators, and outreach workers (Figure 1) requiring a level of autonomy to be able to challenge poor services and care. By understanding the Armed Forces experience and perspective, acknowledging and addressing challenges, optimizing service delivery, and developing essential skills and attributes, the AFCSPLW service can be tailored to meet the unique needs of the AFC population more effectively. This study contributes to the advancement of social prescribing services for the Armed Forces Community and offers valuable guidance for further improvements and nationwide scaling of the AFCSPLW model.

## Strengths and limitations

The study’s mixed-method design allowed for a comprehensive exploration of the AFCSPLW model’s impact, enhancing understanding of its effectiveness. A diverse range of stakeholders participated, enhancing the study’s credibility.

The study addresses a critical gap in knowledge, providing valuable insights into AFC social prescribing model and the AFC health and well-being. However, the two-year timeframe may not fully capture the long-term impact of the AFCSPLW model. Longer follow-up periods would offer a more comprehensive understanding.

While the study used self-report measures, potentially subject to response biases, the overall results indicate the beneficial impact of AFCSPLWs without causing harm. Further research could explore other contributing factors.

The follow-up data collection period varied for different participants. However, the specific time frame between the initial consultation and the last consultation was not shared and in the interviews was indicated that the most common one to one support was between 2–4 weeks, but for some service users was few months. This lack of standardized timing for follow-up consultations introduces variability in the data collection process, potentially influencing the interpretation of the results.

Advocacy, navigation and connections were the keys, with AFC Social Prescribers acting as a link between primary care, third sector and professional networks. This allowed service users to have a voice in settings or meetings where they were not necessarily present. Effective relationships between various sectors and primary care services were crucial, but they take time to develop. Nurturing these relationships is vital for the effectiveness of future social prescribing interventions.
